# Supplementary material for: Identification of limb-specific Lmx1b auto-regulatory modules with Nail-patella syndrome pathogenicity
Source: Nat Commun. 2021 Sep 20;12:5533. doi: 10.1038/s41467-021-25844-5 (PMC8452625; doi:10.1038/s41467-021-25844-5)
Supplement: Supplementary file 1 — Supplementary Information [file 41467_2021_25844_MOESM1_ESM.pdf]

Supplementary Material

Supplementary Table 1

Description	Symbol	GenBank	11.5 FC	12.5 FC	13.5 FC
Lmx1b	*Lmx1b*	NM_001174146.2	15.63	5.68	3.71

The fold change (FC) between normal and *Lmx1b* KO mice at embryonic day (e) 11.5, e12.5 and e13.5 is shown in the table above. The truncated *Lmx1b* mRNA present in *Lmx1b* KO mice is identified by the probes used for gene array anlaysis. In the presence of functional Lmx1b (normal mice), there is a 5.68 fold increase in its own expression when compared to the *Lmx1b* KO mice at e12.5. This data was extracted from the gene array reported by Feenstra et al., 2012[1] GSE34732

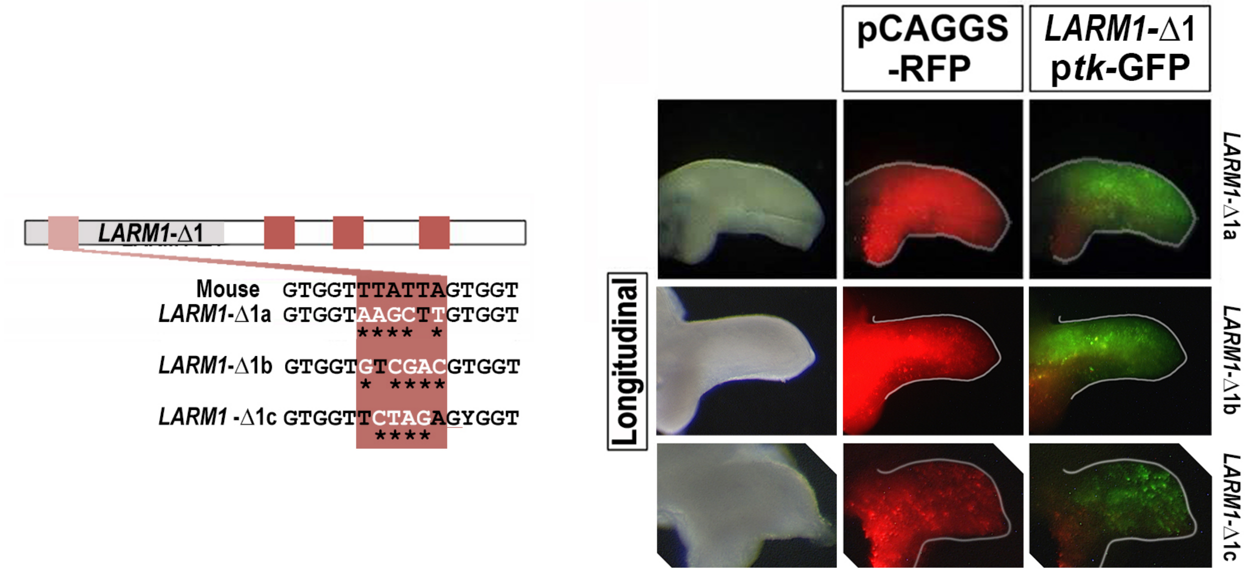

Supplementary Figure 1. Site directed mutagenesis of the 5' element of *LARM1* showing activity extending into the ventral limb mesoderm.

The illustration of *LARM1* is depicted on the left. The silencer region (*LARM1s)* is shaded grey and the 4 Lmx1b binding sites are shaded clay-red according to the TMATWA consensus DNA binding motif for Lmx1b[2]. Below the illustration, the sequence for the Lmx1b binding site within the *LARM1s* region (*LARM1-1)* is displayed in the shaded clay-red column and the nucleotide changes disrupting the Lmx1b binding site for each of the different constructs is presented below the normal sequence. On the right, three alternative disruptions of the Lmx1b binding site showing extension of activity into the ventral mesoderm in chick wing buds 48 hrs after electroporation. The bright-field view show the limb morphology. The RFP fluorescence (red) and GFP fluorescent (green) images of the electroporated limb buds show transfection efficiency and enhancer activity, respectively. *LARM1-Δ1a* is also present in Figure 2D.

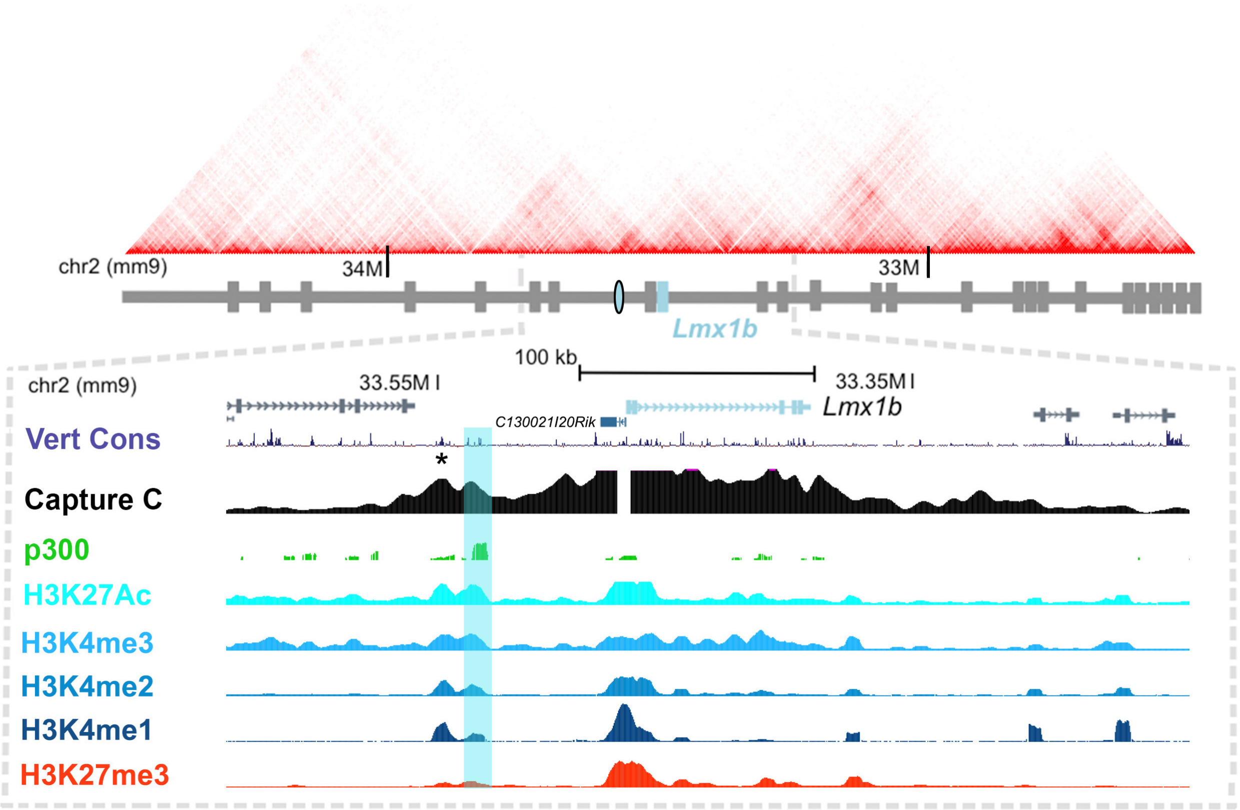

Supplementary Figure 2. *Lmx1b* regulatory landscape

On top the Hi-C interaction profile at 5kb resolution derived from mouse ESCs depicting the interactions in the *Lmx1b* locus and nearby genomic regions[3]. The annotated genes in the region are indicated in grey with *Lmx1b* in light blue and the *LARM* region marked by light blue oval. Below, a zoom in of the genomic region flanked by grey dotted lines containing potential regulatory regions according to Capture-C experiments using the *Lmx1b* promoter as viewpoint[4] together with ChIP-seq profiles for p300[5], H3K4me2[6], H3K27a, H3K3me3, H3K3me1 and H3K27me3 of e11.5 mouse limbs[4]. The *LARM* region is highlighted in blue. The promoter is bidirectional and transcribes a long non-coding RNA (C130021120Rik) from the opposite strand[7,8]. The asterisk indicates an additional potential CRM found upstream of LARM2 within introns and exons of the 9430024E24Rik gene[7]. This conserved region overlaps several chromatin-associated marks indicative of active regulation.

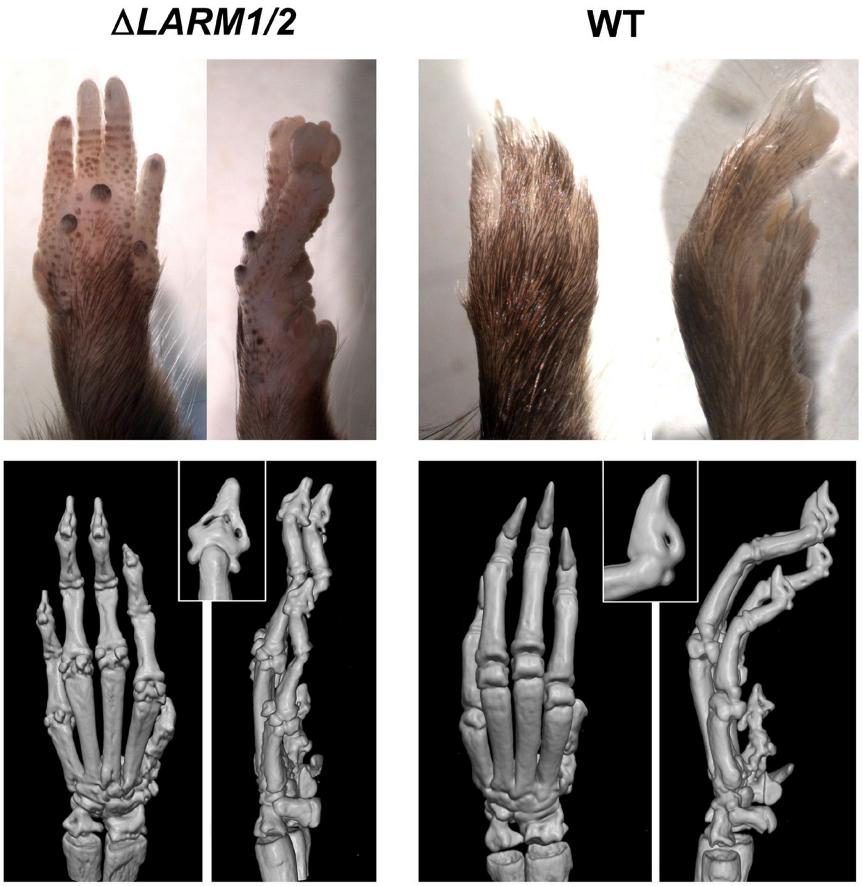

Supplementary Figure 3. Mice lacking the *LARM* region exhibit a ventral-ventral limb phenotype

Dorsal and lateral gross morphology (top) and microCT (bottom) of forelimbs from 6 weeks-old Δ*LARM1/2* (left) and wild type (WT - right) mice. Note bi-ventral Limbs in the mutant. Inset: detail of the distal phalanx (animals examined - Δ*LARM1/2* n=3, Wild type (WT) n=2)**.**

Supplementary Figure 4. The requirement for *LARM1/2* is limb-specific

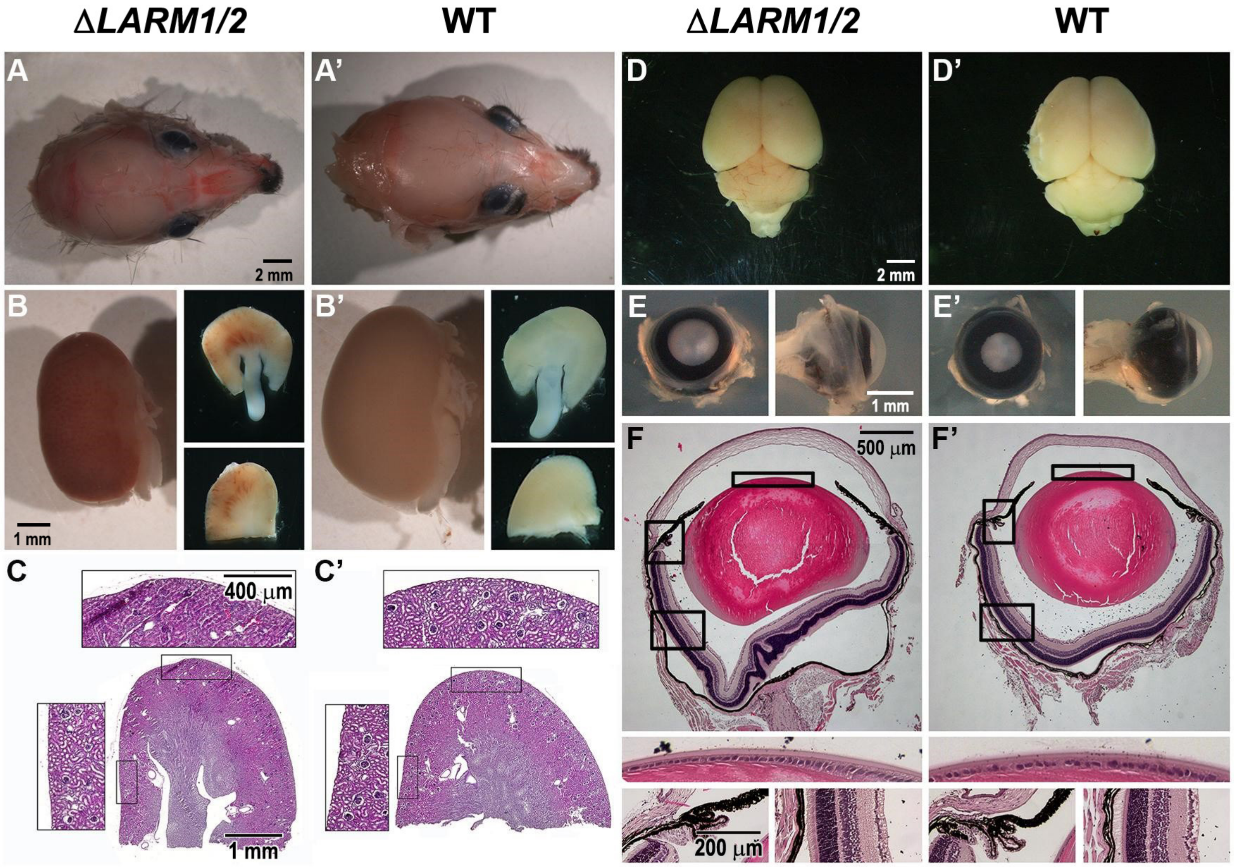

Gross morphology of the calvaria (A-A´), kidney (B-B'), brain (D-D´), and eye (E-E´). Histologic sections of the kidney (C-C´) and the eye (F-F´) stained with Hematoxylin eosin (insets show boxed regions magnified). No overt phenotype in any of the other organs affected in *Lmx1b* knockout mice is present in animals lacking *LARM*1/2 (animals examined - Δ*LARM1/2* n=3, Wild type (WT) n=2).

Supplementary References

1. Feenstra, J.M. *et al.* Detection of genes regulated by Lmx1b during limb dorsalization. *Dev.Growth Differ.* **54**, 451-462 (2012).
2. Haro, E. *et al.* Lmx1b-targeted cis-regulatory modules involved in limb dorsalization. *Development* **144**, 2009-2020 (2017).
3. Bonev, B. *et al.* Multiscale 3D Genome Rewiring during Mouse Neural Development. *Cell* **171**, 557-572.e24 (2017).
4. Andrey, G. *et al.* Characterization of hundreds of regulatory landscapes in developing limbs reveals two regimes of chromatin folding. *Genome Res* **27**, 223-233 (2017).
5. Visel, A. *et al.* ChIP-seq accurately predicts tissue-specific activity of enhancers. *Nature.* **457**, 854-858 (2009).
6. DeMare, L.E. *et al.* The genomic landscape of cohesin-associated chromatin interactions. *Genome Res* **23**, 1224-34 (2013).
7. Gyurján, I., Sonderegger, B., Naef, F. & Duboule, D. Analysis of the dynamics of limb transcriptomes during mouse development. *BMC Dev Biol* **11**, 47 (2011).
8. Taher, L. *et al.* Genome-wide identification of conserved regulatory function in diverged sequences. *Genome Res* **21**, 1139-49 (2011).
