# Supplementary material for: Identification of limb-specific Lmx1b auto-regulatory modules with Nail-patella syndrome pathogenicity
Source: Nat Commun. 2021 Sep 20;12:5533. doi: 10.1038/s41467-021-25844-5 (PMC8452625; doi:10.1038/s41467-021-25844-5)
Supplement: Supplementary file 3 — Description of Additional Supplementary Files [file 41467_2021_25844_MOESM3_ESM.pdf]

Description of Additional Supplementary Files

File name: Supplementary Data 1
Description: This Microsoft Excel file contains the separate sheets for the site directed mutagenesis sequence information for each of the reported constructs; the gRNA sequences used for generating the CRISPR-cas9 knockouts; the primers used for knockout identification; the primers used for NPS patients and the total number of electroporations for each reported experimental condition.

File name: Supplementary Data 2
Description: This pdf contains additional images of each enhancer activity experiment including the transgenic studies.

File name: Supplementary Data 3
Description: This Microsoft Excel file contains the source data for the RT-qPCR of dLARM12 and WT hindlmbs reported in the manuscript in Figure 3i
