# Supplementary figures and images for: Identification of limb-specific Lmx1b auto-regulatory modules with Nail-patella syndrome pathogenicity
Source: Nat Commun. 2021 Sep 20;12:5533. doi: 10.1038/s41467-021-25844-5 (PMC8452625; doi:10.1038/s41467-021-25844-5)

Supplementary Data 2

Additional Images of Each Enhancer Activity Experiment

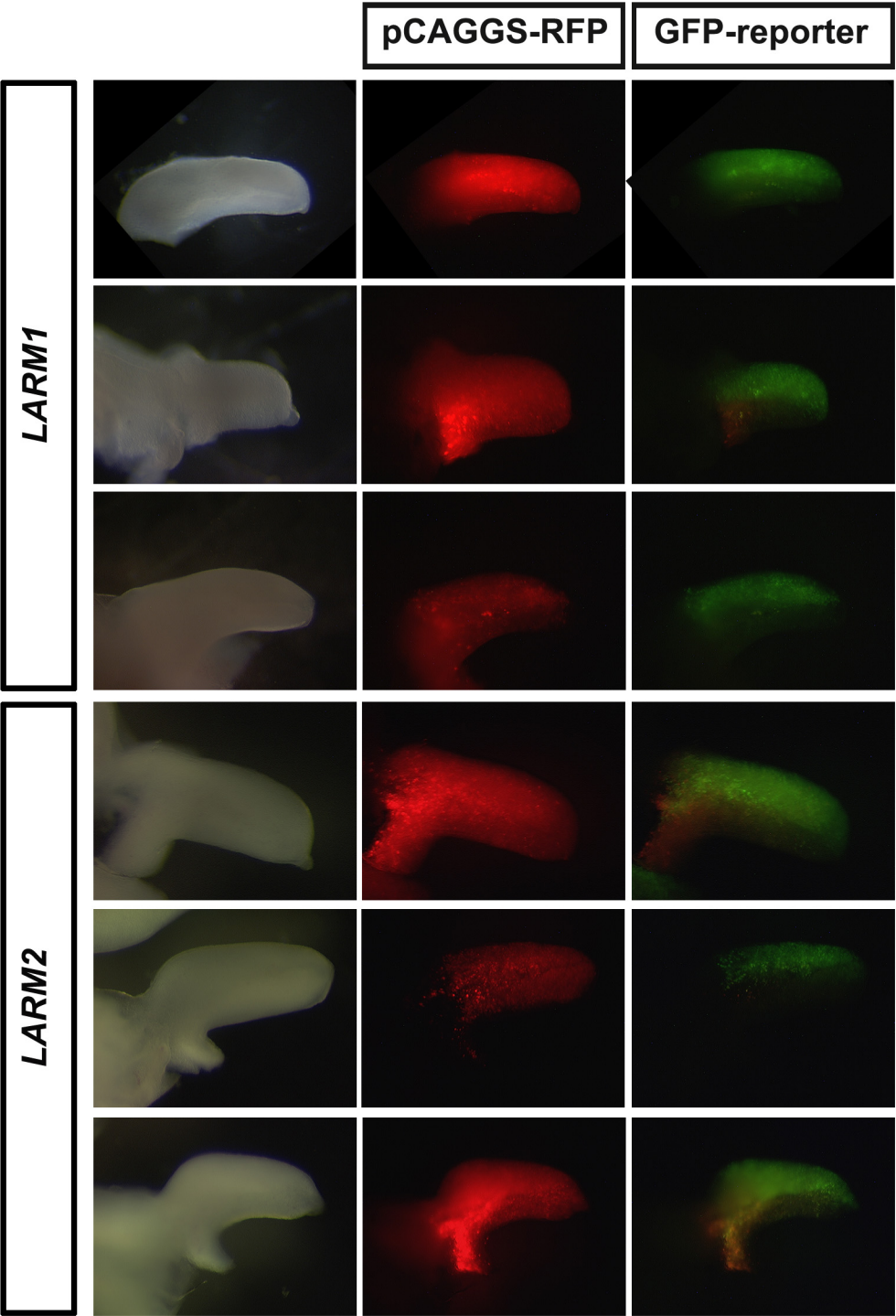

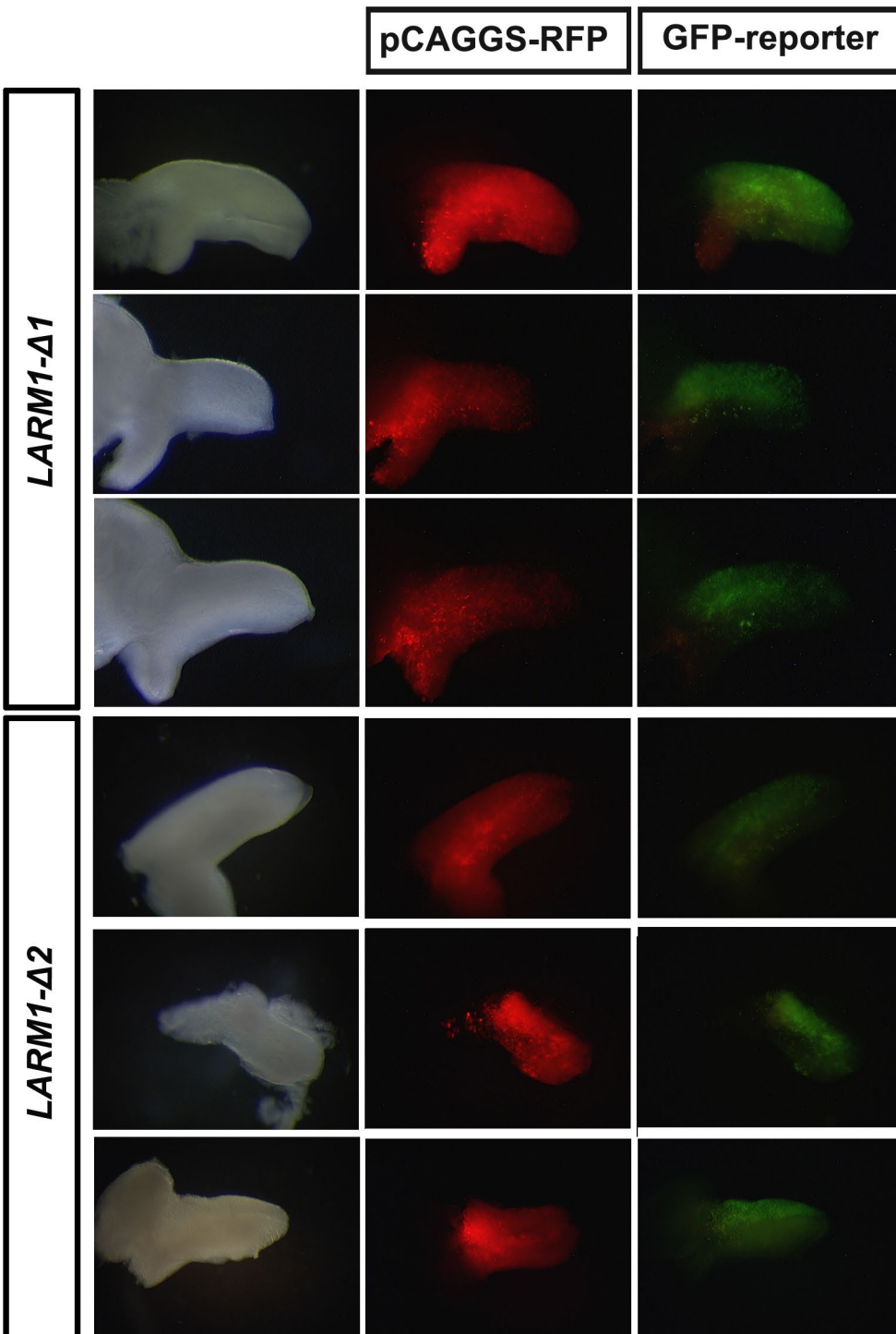

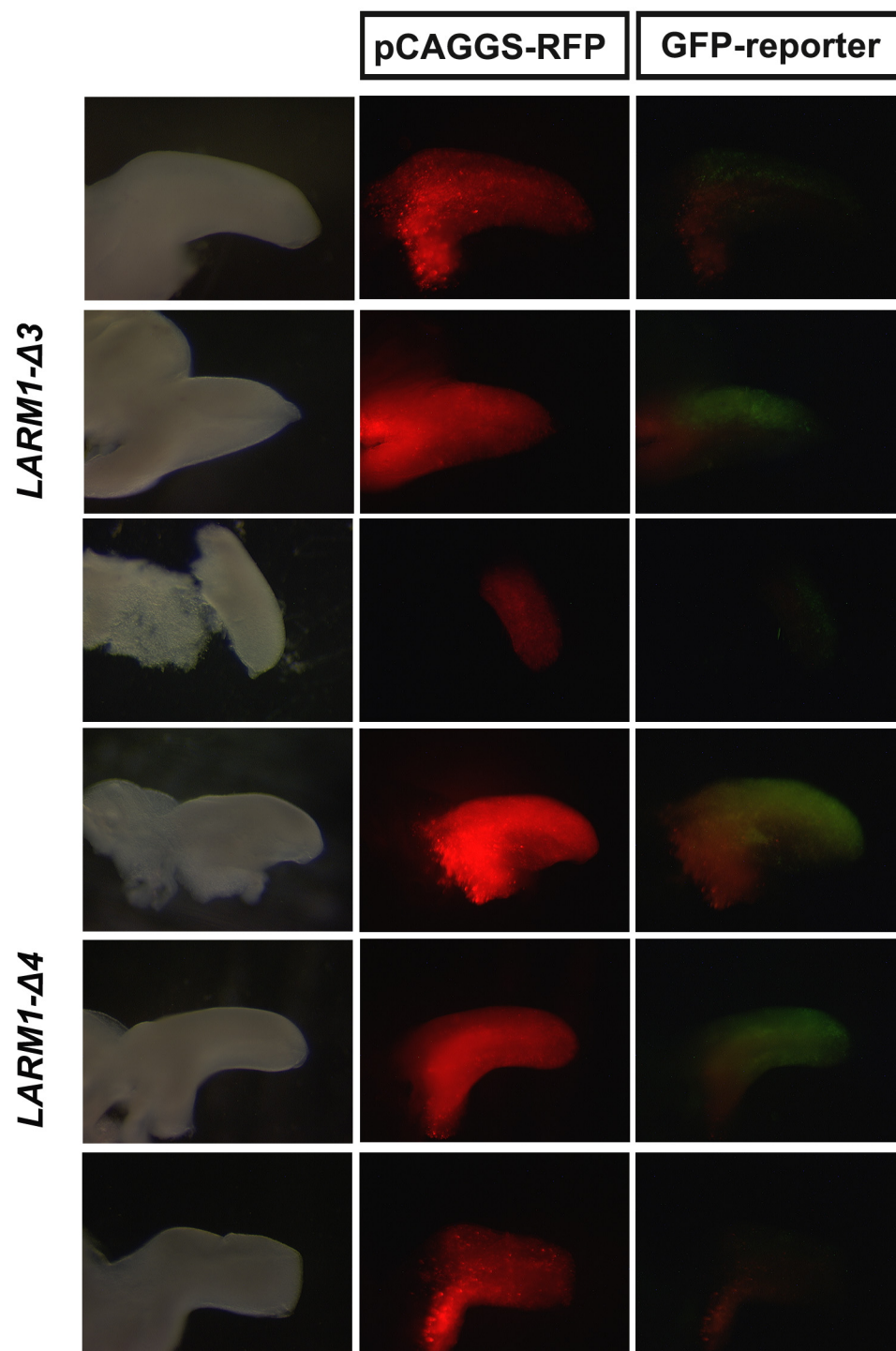

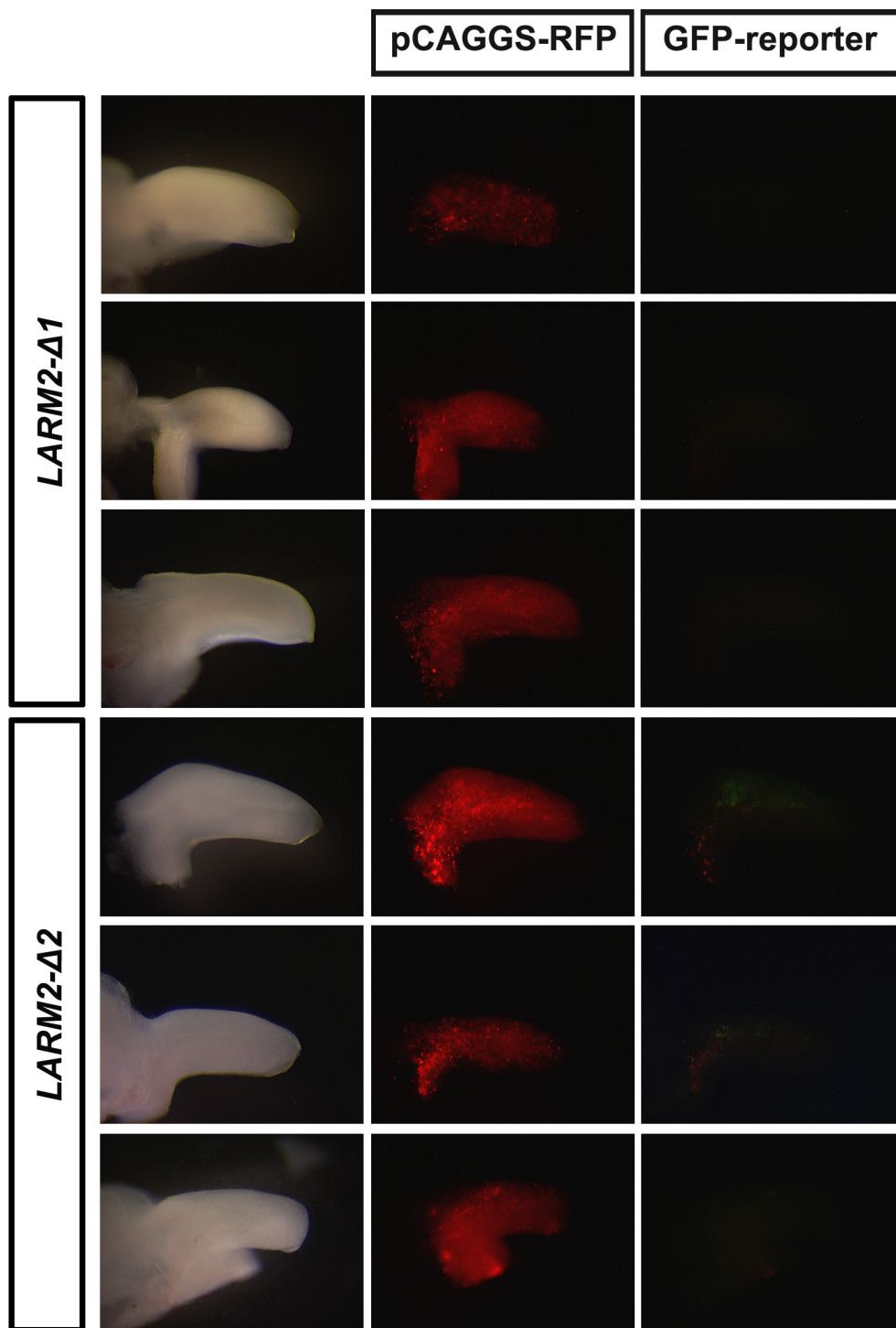

**LARM1/2**

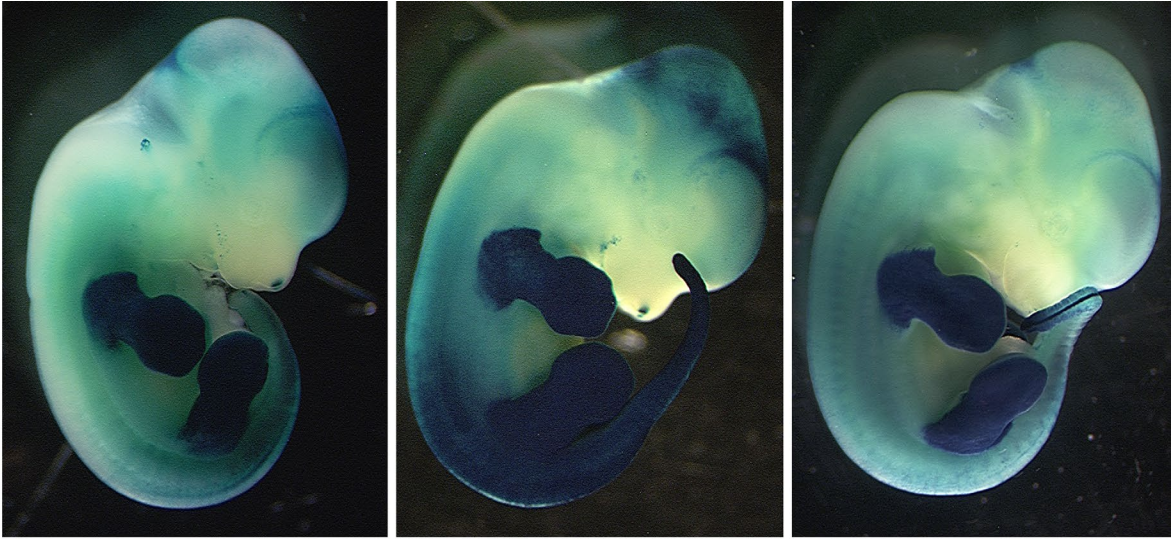

**LARM1**

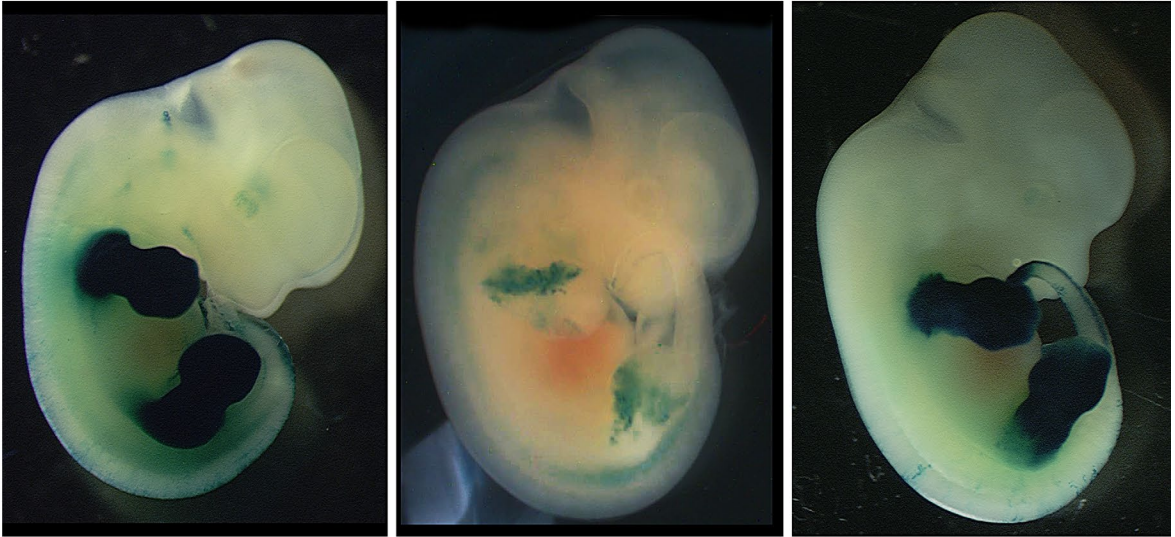

**LARM2**

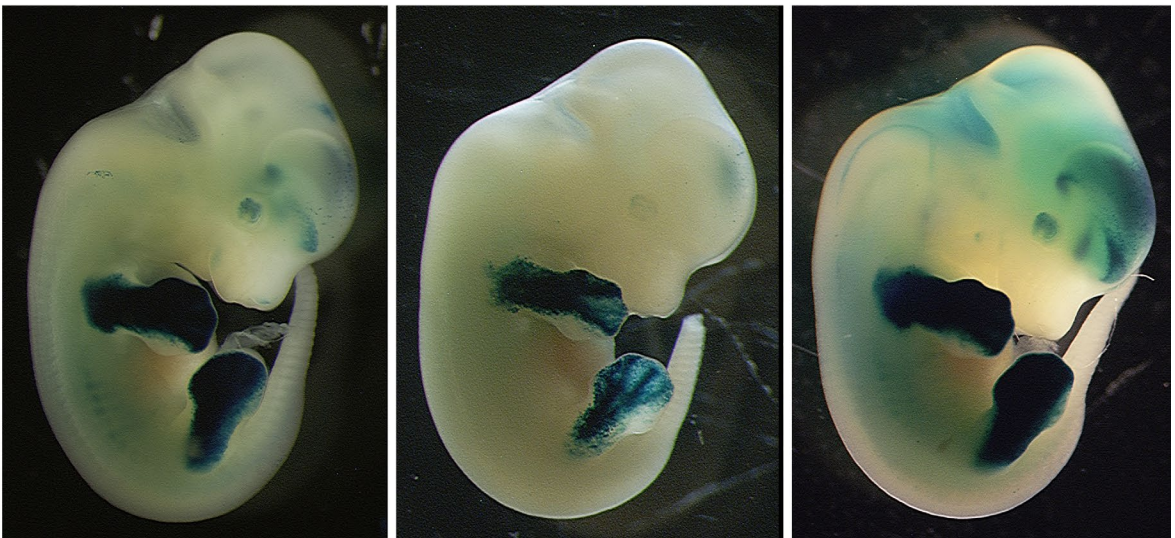

Supplement: Supplementary file 5 — Supplementary Data 2 [file 41467_2021_25844_MOESM5_ESM.pdf]
