# Supplementary material for: Identification of limb-specific Lmx1b auto-regulatory modules with Nail-patella syndrome pathogenicity
Source: Nat Commun. 2021 Sep 20;12:5533. doi: 10.1038/s41467-021-25844-5 (PMC8452625; doi:10.1038/s41467-021-25844-5)
Supplement: Supplementary file 7 — Reporting summary [file 41467_2021_25844_MOESM7_ESM.pdf]

nature research

Corresponding author(s): Kerby C. Oberg, MD, PhD

Last updated by author(s): August 16, 2021

Reporting Summary

Nature Research wishes to improve the reproducibility of the work that we publish. This form provides structure for consistency and transparency in reporting. For further information on Nature Research policies, see our Editorial Policies and the Editorial Policy Checklist.

Statistics

For all statistical analyses, confirm that the following items are present in the figure legend, table legend, main text, or Methods section.

n/a	Confirmed	
☐	☒	The exact sample size (n) for each experimental group/condition, given as a discrete number and unit of measurement
☐	☒	A statement on whether measurements were taken from distinct samples or whether the same sample was measured repeatedly
☐	☒	The statistical test(s) used AND whether they are one- or two-sided<br>*Only common tests should be described solely by name; describe more complex techniques in the Methods section.*
☒	☐	A description of all covariates tested
☒	☐	A description of any assumptions or corrections, such as tests of normality and adjustment for multiple comparisons
☒	☐	A full description of the statistical parameters including central tendency (e.g. means) or other basic estimates (e.g. regression coefficient) AND variation (e.g. standard deviation) or associated estimates of uncertainty (e.g. confidence intervals)
☒	☐	For null hypothesis testing, the test statistic (e.g. F, t, r) with confidence intervals, effect sizes, degrees of freedom and P value noted<br>*Give P values as exact values whenever suitable.*
☒	☐	For Bayesian analysis, information on the choice of priors and Markov chain Monte Carlo settings
☒	☐	For hierarchical and complex designs, identification of the appropriate level for tests and full reporting of outcomes
☒	☐	Estimates of effect sizes (e.g. Cohen's d, Pearson's r), indicating how they were calculated

Our web collection on statistics for biologists contains articles on many of the points above.

Software and code

Policy information about availability of computer code

Data collection	Digital images of fluorescence microscopy (using a Leica MZ8 dissecting micoscope) are acquired using a Sony cat's eye camera (DC-5000) into Adobe Photoshop (version 6.0, acquisition; version 2020, compilation). The skeletal micro Computed Tomography (CT) are scanned with Skyscan1172 at 40 kV, 100 µA, and 27.03 µm pixel resolution and subsequently reconstructed using the NRecon reconstrution software (Ver 1.6.10.2) and compiled with the CTvox version 3.3.1volume rendering software. No unreported custom computer code or algorithm was used to collect data or generate results reported in this paper.
Data analysis	Microsoft Excel 2016 is used for tabulated data. No unreported custom computer code or algorithm was used to collect data or generate results reported in this paper

For manuscripts utilizing custom algorithms or software that are central to the research but not yet described in published literature, software must be made available to editors and reviewers. We strongly encourage code deposition in a community repository (e.g. GitHub). See the Nature Research guidelines for submitting code & software for further information.

Data

Policy information about availability of data

All manuscripts must include a data availability statement. This statement should provide the following information, where applicable:

- Accession codes, unique identifiers, or web links for publicly available datasets
- A list of figures that have associated raw data
- A description of any restrictions on data availability

The data that support the findings of this study are included within the manuscript or in the supplemental data. The copy number variation data (4.5 kb heterozygous deletion) described in this study has been reported in the Decipher database under the ID#433715 (https://www.deciphergenomics.org/). The data generated in Fig. 3I of this study are provided in the Source Data file provided with this paper as supplemental data. Additional embryos from the enhancer assays

represented in Fig. 2 and the transgenic data shown in Fig. 4 are provided in the supplemental data Figure S5.
Publically available datasets were also used in this study:
- Lmx1b gene array data, e12.5 limbs, is available through the Gene Expression Omnibus (GEO) database, (http://www.ncbi.nlm.nih.gov/geo/) under accession number GSE34732
- Limb ChIP-seq data are available under the GEO database accession numbers: GSE84064 for Lmx1b (https://www.ncbi.nlm.nih.gov/geo/query/acc.cgi?acc=GSE84064), GSE42413 for H3K27Ac (https://www.ncbi.nlm.nih.gov/geo/query/acc.cgi?acc=GSE42413), GSE13845 for p300 (https://www.ncbi.nlm.nih.gov/geo/query/acc.cgi?acc=GSE13845), GSE42237 for both H3K27me & H3K4me (https://www.ncbi.nlm.nih.gov/geo/query/acc.cgi?acc=GSE42237).
- RNA Pol II and Med12 ChIP-Seq datasets are available as supplemental datasets S1 and S2 from Berlivet and coworkers13.
- Capture-C data was mined from previous published work deposited in the GEO database accession number GSM2251518.

Field-specific reporting

Please select the one below that is the best fit for your research. If you are not sure, read the appropriate sections before making your selection.

☒ Life sciences ☐ Behavioural & social sciences ☐ Ecological, evolutionary & environmental sciences

For a reference copy of the document with all sections, see nature.com/documents/nr-reporting-summary-flat.pdf

Life sciences study design

All studies must disclose on these points even when the disclosure is negative.

Sample size	For quantitative data, three-four biological replicates were analyzed for each genotype, with 3 technical replicates per sample. We anticipated at least a 2 fold difference between controls and knockout animals based on the phenotype and even with a standard deviation that was 20% of the mean, we expected that 3 embryos would detect this difference with statistical significance and our qPCR data returned a p value of 6.7 x10e-5. For qualitative analysis of enhancer activity, the technique requires injection of DNA followed by electroporation, which can compromise survival in these young embryos. Positive and negative controls are run with each experiment for comparison. Only limbs in surviving chicks that exhibiting adequate transfection efficiency (adequate RFP expression and coverage) were further analyzed for experimental activity. Since the experiments were reporting on the activity pattern, the experimental numbers recovered were considered sufficient if the same pattern of activity was demonstrated in limbs from experiments on 3 separate days that had adequate controls. LARM1 and LARM2 were used as positive controls in experiments with site directed mutagenesis. All sample numbers and outcomes are listed in supplemental table S2 and additional image data of enhancer activity studies are supplied in supplemental Figure S5.
Data exclusions	For qualitative data, if electroporated limbs in surviving chicks did not exhibit adequate transfection efficiency, they were not further analyzed.
Replication	Noted above. Experiments on at least three separate days or 3-4 biologic replicates were used to confirm reproducibility.
Randomization	For the chicken limb bioassay, staged eggs are sequentially injected and electroprated. A single glass pulled micro needle is used for the injection of DNA for a specific experimental group. When the DNA is depleted, DNA for another experimental group is loaded in a new needle for injection. The eggs are randomly sorted into cohorts prior to injection. Replicate experiments were performed on different days to account for random variations in techniques or reagents. In the knockout mice studies, the experimental groups are determined by the presence of a mutation so are not random. However, both the mice and chickens used for these studies are from specific strains reducing genetic variability.
Blinding	Blinding is most helpful when qualitative assessments are made based on treatment and collected as part of the data to be analyzed and thus knowing the treatment can bias the assessment and outcome data. In this report, data meeting the qualitative transfection criteria are reported and the data presented to allow the reader to determine whether they agree with the authors assessments. Nevertheless, we did ask other members of our lab that did not perform the experiments to offer their interpretation of the data without knowing the experimental conditions as an internal control to confirm our interpretations.

Reporting for specific materials, systems and methods

We require information from authors about some types of materials, experimental systems and methods used in many studies. Here, indicate whether each material, system or method listed is relevant to your study. If you are not sure if a list item applies to your research, read the appropriate section before selecting a response.

Materials & experimental systems

n/a	Involved in the study
x	Antibodies
	x Eukaryotic cell lines
x	Palaeontology and archaeology
	x Animals and other organisms
	x Human research participants
x	Clinical data
x	Dual use research of concern

Methods

n/a	Involved in the study
x	ChIP-seq
x	Flow cytometry
x	MRI-based neuroimaging

Eukaryotic cell lines

Policy information about cell lines

Cell line source(s)	Neuroblastoma (N2a) cells were used by Sigma-Aldrich to validate gRNAs before returning them to us.
Authentication	By Sigma-Aldrich
Mycoplasma contamination	Unknown
Commonly misidentified lines (See ICLAC register)	NA

Animals and other organisms

Policy information about studies involving animals; ARRIVE guidelines recommended for reporting animal research

Laboratory animals

Mice used for generation of delta-LARM1/2, delta-LARM1 and delta-LARM1 mutant lines:

Mice were housed under appropriate lighting level in standard 12-h light/12-h dark cycles, 21-23ºC ambient temperature and 55% (+-10%) humidity. Ad libitum Food (standard chow) and fresh water was freely accesible (ad libitum). Scattered food (e.g. sunflower seeds) was also provided in substrate for stimulation. Cleaning protocols were designed balancing hygiene with stress avoidance. Housing was settle in stable, compatible groups according to age, sex, reproductive condition, and prior group housing experience. Cages selected to provide enough space (290 cm2, minimum 70-100cm2 per animal depending on the weight) and height (12 cm) to allow exploration and play. Cage floors were covered with at l least 1cm of dust-free woodchip hygienic substrate. Enrichment materials (cardboard tubes, hard pellets etc…) and nesting material (soft paper) when pertinent were included in the cages.

All the following procedures were carried out by trained specialized personnel in a specialized surgery room. After surgery all animals were placed in a quite area, provided with a warm blanket and closely monitored until ambulatory. Then, the wound was inspected daily until complete heal.

Male vasectomy: Vasectomized males needed to engender pseudopregnancy in mature female mice were generated following standard procedures. Ten CD1 vasectomized males were used in this study.

Donor females: 10 CBAC56BL6 (F1) hybrid females (4-6 weeks-old) were used for each CRISPR-Cas9 modification. The females were subject to superovulation with PMSG (day 1) and hCG (day 3) and crossed with C56BL6 males to obtain the 0.5 dpc zygotes.

Foster mothers: CD1 female mice (8-12 weeks old) were mated with vasectomized males to induce pseudopregnancy. For each CRISPR modification, the electroporated zygotes were transferred into the oviductal infundibulum of 4 foster mothers. Offspring of these females were genotyped to select the founder (F0) that was crossed back with C56BL6 to establish the line that was maintained in hetorozygosis.

Phenotypic characterization: Crosses between N2 heterozygous mice carrying the CRISPR-Cas9 induced modification were set up to obtain homozygous embryos. At least 5 homozygous embryos were analyzed per stage (e12.5, P1, 3-weeks and 6 weeks-old). No differences were scored between males and females.

Transgenic mice: Transgenic mouse generation and staining were performed at Cyagen Inc, (Sanat Clara, CA) and sent to us for analysis.

Chicken embryos for electroporation studies: Fertilized Gallus gallus chick eggs were used for embryonic chicken experiments. Sex is not determined in the embryos which are at Hamilton and Hamburger stage 14 (54 hrs after laying) for the limb bioassay.

Wild animals	This study did not involve wild animals
Field-collected samples	This study did not involve samples collected from the field

Ethics oversight	All animal procedures were reviewed and approved by the Loma Linda University Institutional Animal Care Use Committee (IACUC) or by the Bioethics Committee of the University of Cantabria and performed according to the EU regulations, animal welfare and 3R principles

Note that full information on the approval of the study protocol must also be provided in the manuscript.

Human research participants

Policy information about studies involving human research participants

Population characteristics	Patients diagnosed with Nail Patella Syndrome that did not have a coding sequence mutation.
Recruitment	We have obtained informed written consent from all participants for diagnostic genetic analyses. These patients are not in a research study, rather to determine if genetic variants that might explain their condition could be identified. No identifying information or identifiable images of the human participants are included.
Ethics oversight	The study was reviewed by the Institutional Ethics Committee of the University of Lille and was found to be in accordance with the criteria set by the Declaration of Helsinki.

Note that full information on the approval of the study protocol must also be provided in the manuscript.
